# Reliability of a novel dynamic test of postural stability in high-level soccer players

**DOI:** 10.1016/j.heliyon.2021.e06647

**Published:** 2021-04-21

**Authors:** Paul E. Beelen, Ricardo Okhuijsen, Maarten R. Prins, Arnold Huurnink, Tim Hordijk, Christiaan Kruiswijk, Edwin A. Goedhart, Peter van der Wurff, Peter A. Nolte, Jaap H. van Dieën, Idsart Kingma

**Affiliations:** a Vrije Universiteit, Department of Human Movement Sciences, Amsterdam Movement Sciences, Amsterdam, the Netherlands; b Military Rehabilitation Centre ‘Aardenburg’, Research and Development, Doorn, the Netherlands; c Sports Medicine Centre of the Royal Netherlands Football Association/FIFA Medical Centre of Excellence, Zeist, the Netherlands; d Spaarne Gasthuis Hospital, Hoofddorp, Noord-Holland, the Netherlands

**Keywords:** Postural stability, Perturbation, Time To Stabilization, Center of Pressure speed

## Abstract

Postural stability of athletes is commonly tested with single-leg stance (SLS) tests. However, for this population, these tests are insufficiently challenging to achieve high sensitivity. Therefore, a new dynamic SLS test based on standardized translational surface perturbations was developed. This study aimed to assess reliability, sensitivity to learning effects, and internal and concurrent validity of this novel test.

Healthy soccer players (21 females, 21 males) performed 2 test sessions. Each session consisted of 2 trials. For one trial, the participant performed a 30-seconds, unperturbed SLS on each leg, followed by 12 platform perturbations per leg.

Intraclass Correlation Coefficients (ICC) and correlations between outcomes were calculated for the Center of Pressure speed (CoPs) and Time To Stabilization (TTS). ANOVA was used to assess learning effects. CoPs and TTS showed a fair reliability between sessions (ICC = 0.73–0.76). All variables showed improvement over time within and between sessions (all p < 0.01) and were moderately correlated with CoPs during unperturbed SLS (r = 0.39–0.56).

Single-leg dynamic postural stability testing through standardized horizontal platform perturbations yielded sufficiently reliable CoPs and TTS outcome measures in soccer players. The moderate correlations with unperturbed SLS support concurrent validity, but also indicates that the new test captures aspects of postural stability that differ from the conventional, unperturbed SLS test.

## 1 Introduction

Traditionally, in sports, postural stability of the lower extremity is evaluated using single-leg stance tasks on a solid or an unstable surface [1, 2, 3]. The instruction is to stand as still as possible, and less center of mass movement is assumed to reflect better performance. Lower sway during the single-leg stance (SLS) test is associated with a higher level of sports performance [1, 4], better performance on various agility tests [5], and reduced ankle sprain risk [6], while higher sway is related to impairments after concussion [7, 8]. The SLS thus appears to provide relevant information about the sensorimotor control system, which coordinates the timing and magnitude of corrective motor actions [9] subserving postural stability, which is the ability to control the body position in space for the purpose of movement and balance [10].

Coordination of corrective motor actions is considered particularly important to control the body during demanding, rapidly changing, or unexpected movement in sports such as soccer [5]. However, the SLS lacks all these aspects of motor behavior. Therefore, more challenging balance tests have been developed to improve detection of sport-specific balance impairments [11, 12, 13]. Most commonly used are balancing on a wobble board [14], or more sports-specific tests involving hopping or drop-jumps, where the focus is on unipedal landing [15, 16]. As a proxy for center of mass control, the Center of Pressure (CoP) trajectory (e.g. amplitude and speed) and horizontal ground reaction forces (HGRF) have been shown to be valid and reliable outcome measures [17, 18, 19]. Another commonly used outcome measure in these dynamic balance tests is the Time To Stabilization (TTS) [16, 20, 21]. The TTS has been used in several ways, but a robust and valid measure is the time it takes for the vertical ground reaction force to return within the preset threshold of 5% body weight. Drop-jump outcomes were found to be moderately related to SLS outcomes [18, 22]. However, drop-jump outcomes showed low reliability, especially for those outcomes reflecting the most dynamic part of the task directly after landing, probably due to the variability in jump height or the difficulty of the test [17, 20, 23, 24, 25]. This will limit the usability of the test to identify individuals with balance impairments.

Recently, devices have been developed to assess the dynamic postural stability. Among these are uni- and omni-axial balance boards [26]. An instrumented unstable uni-axial balance platform was not sufficiently sensitive to detect effects of fatigue after a treadmill run [27]. Moreover, balancing on such balance boards is learned rapidly and learning effects are retained for a long time [28]. A suddenly rotating platform was able to differentiate between participants with Parkinson's Disease and healthy controls, but this difference disappeared after practicing [29].

To date, there is little research on dynamic balance following horizontal perturbations of the upper body relative to the feet, even though this is a key aspect of sensorimotor control during demanding sports and especially contact sports. One study used horizontal translations of the support surface and found a significant effect of low-back pain on postural stability [30]. Platform translations can be adjusted to provide an adequate and safe challenge even for patients with impaired postural stability after a cerebral vascular accident [31]. Building on these observations regarding platform translations and to overcome the aforementioned limitations, a test was developed to provide demanding unexpected standardized perturbations during single-leg stance. For this test outcome measures similar to drop landing can be calculated, but the reliability of such outcome measures is unknown. Therefore, we aimed to analyze the reliability and to assess potential learning effects of dynamic single-leg balance performance following standardized horizontal platform perturbations. Furthermore, to evaluate internal and concurrent validity, the interrelations among these outcome measures, and correlations with respect to static SLS, were determined. We hypothesize that the outcome measures of postural stability assessed by standardized horizontal platform perturbations are sufficiently reliable and that a significant learning effect is present.

## 2 Methods

### 2.1 Participants

42 healthy soccer players were recruited to participate in our study (21 males; mean (range) age 24 (19–36) years; height 182 (170–190) cm; body mass 75 (59–95) kg and 21 females; mean (range) age 23 (16–29) years; height 171 (157–181) cm; body mass 67 (56–80) kg). Participants were recruited through the network of the Royal Dutch Football Association (KNVB). The sample consisted of 16 male indoor professionals, 9 female field professionals, 5 male and 12 female field amateurs (ranging from lower regional to national league). The exclusion criteria were injuries of the lower extremity in the past six months and lower extremity surgery in the past two years and any condition that might interfere with postural stability, such as current lower extremity pain or a neurological disorder. The study was approved by the Human Ethics Committee of the Faculty of Movement and Behavioral Sciences of the Vrije Universiteit in Amsterdam (Ethical Approval ID: ECB-2014-34). This quantitative, cross-sectional study was conducted according to the principles of the Declaration of Helsinki [32]. All participants provided written informed consent before measurements took place.

### 2.2 Equipment

**Figure 1:**
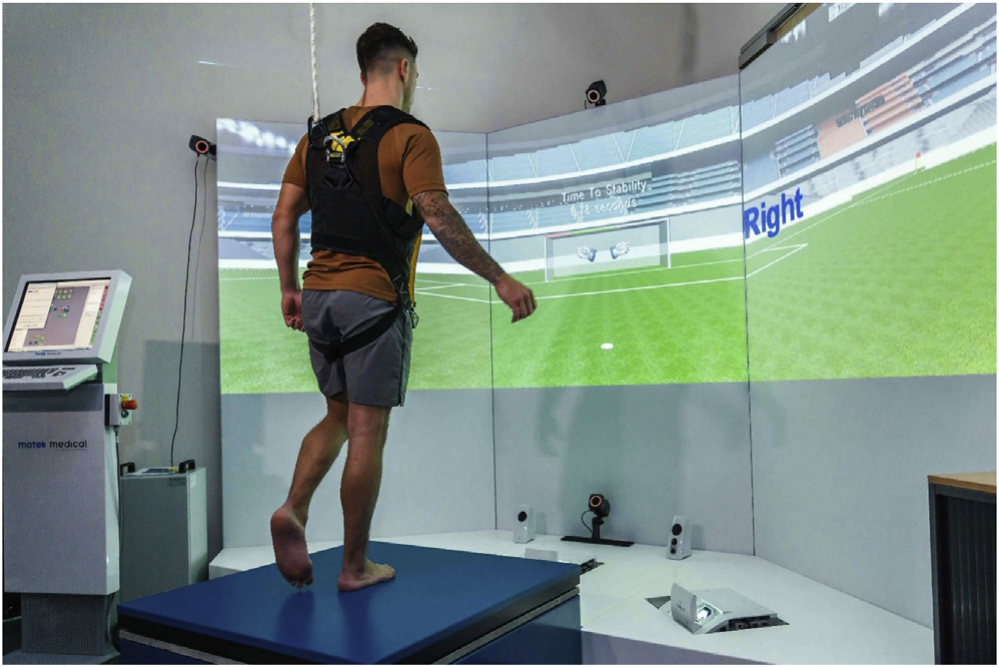
The set-up of the position-controlled movable platform with an integrated force plate and the screen providing real-time visual feedback.

The static and dynamic tests were performed on a position-controlled movable platform (100 × 100 cm) with an integrated force plate, which are part of the Dynamic Stability and Balance Learning Environment (DynSTABLE, Motek Force link BV, Amsterdam, The Netherlands). The force plate measured the CoP and magnitude of the vertical forces. To enhance performance motivation of subjects, real-time visual feedback of CoP speed values was provided to subjects during all trials. This information was provided on a monitor placed in front of the platform, by means of the size of a pair of goalkeeper gloves (larger for higher CoP speed). During the measurements, participants wore a safety harness (Petzl® Newton Fast Jak) suspended overhead to prevent falls, but no weight support was provided (Figure 1).

### 2.3 Procedures

Testing was done at the Sports Medicine Centre (SMC) of the Royal Netherlands Football Association (KNVB). Two test sessions were performed with on average 10 days (range 5–30) in between. Each session comprised two similar trials with a rest period of 5 min in between. Each trial took approximately 5 min. First an unperturbed SLS test was performed, with the instruction to stand as motionless as possible for 30 s. This was first performed standing on the left leg and was repeated standing on the right leg. Subsequently, participants experienced 12 perturbations of single-leg stance per leg by mediolateral translations of the platform at an average speed of 0.32 (SD = 0.01) m/s over a distance of 2 cm. In these perturbation tests, participants switched between legs after 4 perturbations (2 lateral and 2 medial in quasi-randomized order), with the left leg as the initial testing leg. Participants were instructed to regain motionless stance as quickly as possible after each platform perturbation. Participants were tested barefooted and were instructed to focus on the screen and keep their hands in their sides. The time between perturbations varied between 5-8 s.

### 2.4 Data processing and analysis

Force plate data were sampled at 1000 samples/s for 5 s, starting 200 ms after the initiation of the perturbation. These data were analyzed with custom-made software (MATLAB R2013a, The Mathworks, Inc., Natrick, RI, USA). Raw data were bi-directionally filtered with a 4 Hz fourth order (2 × 2^nd^ order) Butterworth low-pass filter at a cut-off frequency of 12 Hz [18, 33].

The first outcome measure was the mean CoP speed (CoPs) calculated as the total CoP path length divided by the measurement time window [1, 9, 18, 34, 35, 36]. This outcome measure was calculated for both the static and dynamic postural stability tests. For the SLS test, the full time window of 30 s was used. For the dynamic postural stability test, a time window of 2 s post-perturbation (starting 200 ms after initiation of the perturbation), which equals about twice the TTS, was used. As the platform perturbations were all in mediolateral direction, CoPs was also calculated in mediolateral (CoPs ml) and anteroposterior direction (CoPs ap) separately.

The second outcome measure was the TTS, which represents the time elapsed from perturbation until the vertical ground reaction force remains within 97.5–102.5% of the body weight for 1 s. For landing tasks, the TTS method is well established, however we used 97.5–102.5% range instead of the commonly used 95–105% range [15, 16, 37], because of the smaller magnitude of the perturbations in our study.

An additional outcome measure was ‘Imbalance’, which is the total number of perturbations after which the subject was unable to remain standing on one leg (e.g. tapping the force plate with the non-standing leg) [20]. Note that the perturbations labeled as ‘Imbalance’ were excluded from CoPs and TTS calculations.

### 2.5 Statistical analysis

Intraclass Correlation Coefficients (ICC) for consistency (single and average) between and within sessions were assessed for static and dynamic postural stability outcomes. The ICC values were classified as poor (ICC <0.70), fair (0.70 ≤ ICC <0.80), good (0.80 ≤ ICC <0.90), and excellent (ICC ≥0.90) [30]. Analyses were performed for the average values of both legs. To compare outcome measures within the tests as well as between dynamic and static tests, Pearson's correlations between all the outcome measures were calculated. To interpret the correlation coefficients, for absolute values of r, 0–0.19 was regarded as very weak, 0.2–0.39 as weak, 0.40–0.59 as moderate, 0.6–0.79 as strong and 0.8–1 as very strong [31]. A repeated measures ANOVA with factors session (sessions 1 and 2) and trial (trials 1 and 2) was used to analyze changes over time in the outcome measures (IBM SPSS Statistics 25). As sex may affect postural stability, sex was added as a between-subject factor in the ANOVA. Huynh-Feldt significance level was set at p < 0.05.

## 3 Results

**Figure 2:**
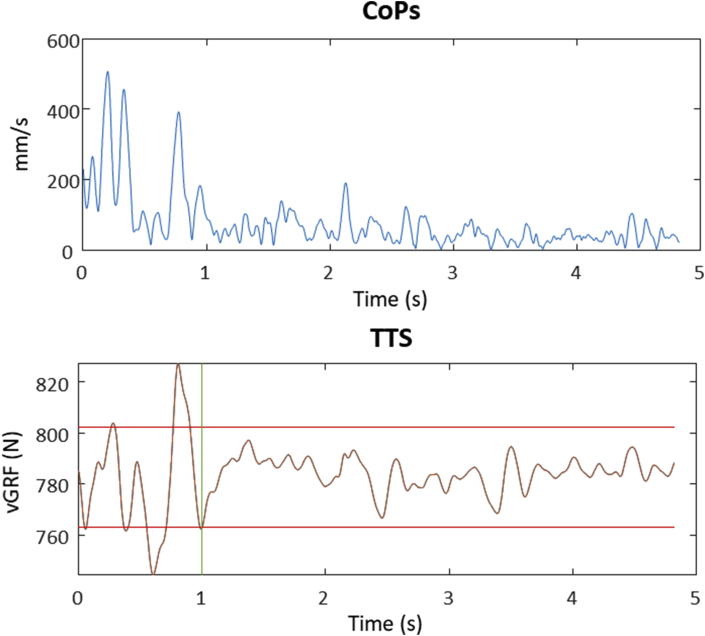
A typical example of the CoPs (mm/s) (above) and the vertical ground reaction force (N) (below) to calculate the TTS after one platform perturbation for one participant. The vertical green line gives an indication of the TTS. The horizontal red lines represent the thresholds (±2,5% body weight). CoPs = Center of Pressure speed, TTS = Time To Stability, vGRF = vertical ground reaction force, N = Newton, mm = millimeter, s = seconds.

Figure 2 shows an example of the CoPs and the vertical ground reaction force in the 5 s after a perturbation of a participant in the first trial.

### 3.1 Reliability

The between-session reliability (ICC single, Table 1) was fair for most outcome measures (0.68–0.76), with the lowest ICC for Imbalance and the highest for TTS. Note that the data of Imbalance are not normally distributed. When using the average value of the two test sessions, the ICC values improved to 0.81–0.87. The reliability within sessions (ICC average, Table 1) was similar between sessions 1 (0.81–0.90) and 2 (0.82–0.94).

**Table 1:** ICC values (95% confidence interval) for all outcome measures. ICCs were calculated between session 1 and session 2 (single and average) and within session 1 and 2 for the average value of both legs.

ICC value	ICC single Between sessions	ICC average Between sessions	ICC average Within session 1	ICC average Within session 2
CoPs static	0.73 (0.56–0.85)	0.85 (0.71–0.92)	0.85 (0.71–0.92)	0.82 (0.66–0.90)
CoPs dynamic	0.75 (0.58–0.86)	0.86 (0.74–0.92)	0.90 (0.82–0.95)	0.91 (0.83–0.95)
CoPs ml	0.74 (0.56–0.85)	0.85 (0.72–0.92)	0.81 (0.65–0.90)	0.89 (0.79–0.94)
CoPs ap	0.75 (0.58–0.86)	0.86 (0.73–0.92)	0.90 (0.82–0.95)	0.91 (0.83–0.95)
TTS	0.76 (0.60–0.87)	0.87 (0.75–0.93)	0.87 (0.76–0.93)	0.92 (0.85–0.96)
Imbalance	0.68 (0.48–0.82)	0.81 (0.65–0.90)	0.89 (0.79–0.94)	0.94 (0.88–0.97)

CoPs = Center of Pressure speed, ml = mediolateral, ap = anteroposterior, TTS = Time To Stabilization.

### 3.2 Validity

Reflecting internal validity, TTS correlated moderately to strongly with CoPs measures (r = 0.52–0.66) and the CoPs correlated very strong with both CoPs ml (r = 0.87) and CoPs ap (r = 0.99). Correlations between imbalance and the other outcome measures were not significant. Reflecting concurrent validity, all outcome measures of the dynamic test (r = 0.39–0.56; Table 2), except for Imbalance, correlated weakly to moderately with the CoPs in the static SLS test.

**Table 2:** Correlations of all the outcome measures for platform perturbations. Correlations ≥0.80 are in bold. Significant correlations (p < 0.05) are indicated with an asterisk.

Correlations	CoPs static	CoPs	CoPs ml	CoPs ap	TTS	Imbalance
CoPs static	**-**	0.54∗	0.56∗	0.50∗	0.39∗	-0.03
CoPs dynamic	0.54∗	-	**0.87∗**	**0.99∗**	0.65∗	-0.04
CoPs ml	0.56∗	**0.87∗**	**-**	**0.80∗**	0.52∗	-0.02
CoPs ap	0.50∗	**0.99∗**	**0.80∗**	**-**	0.66∗	-0.05
TTS	0.39∗	0.65∗	0.52∗	0.66∗	-	-0.22
Imbalance	-0.03	-0.04	-0.02	-0.05	-0.22	-

CoPs = Center of Pressure speed, ml = mediolateral, ap = anteroposterior, TTS = Time To Stabilization.

### 3.3 Learning effects

**Figure 3:**
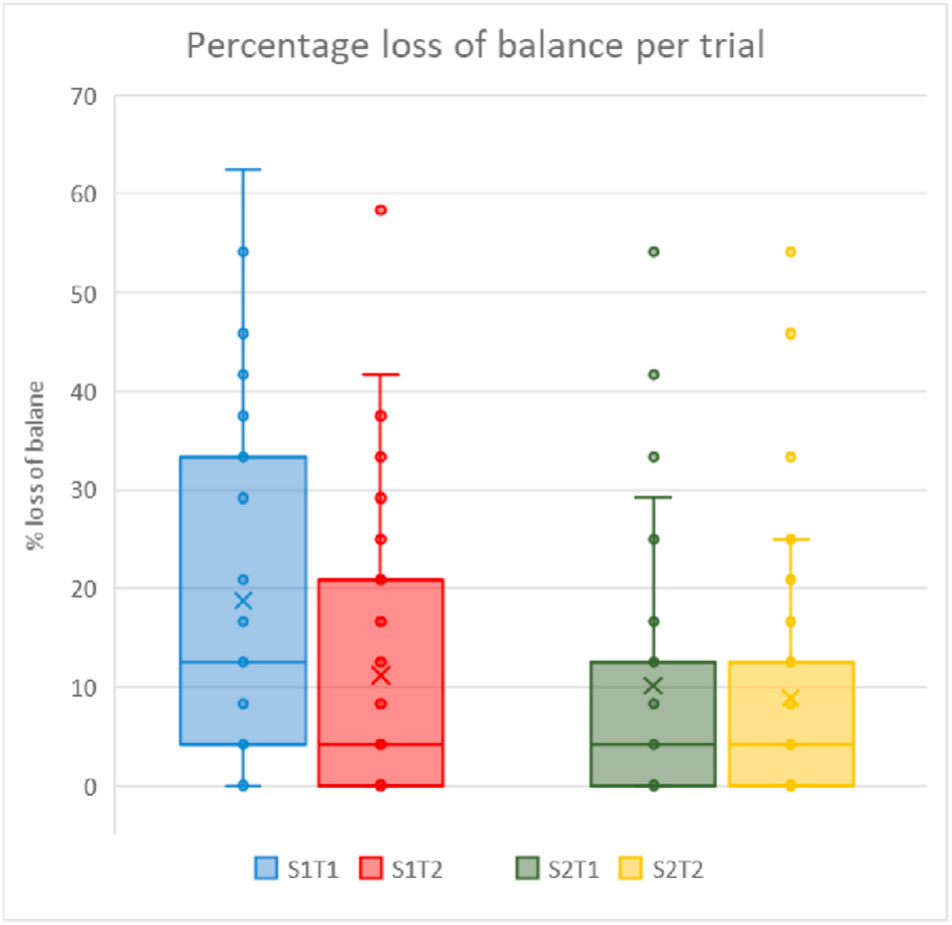
Boxplots of the percentage of trials in which participants lost postural stability. On the X-axis are the four trials divided over two sessions. Quartiles, medians and individual data points (including outliers) are shown for trials 1 and 2 of session 1 and 2. The x represents the mean in a single session. S = Session and T = Trial, with for example S1T1 = Session 1 and Trial 1.

The number of ‘imbalance trials’ in the dynamic tests, which were discarded for TTS and COPs analysis, was on average 12.2% per trial, with more loss of postural stability in Trial 1 of Session 1 compared to all other trials (p < 0.01, Figure 3).

**Figure 4:**
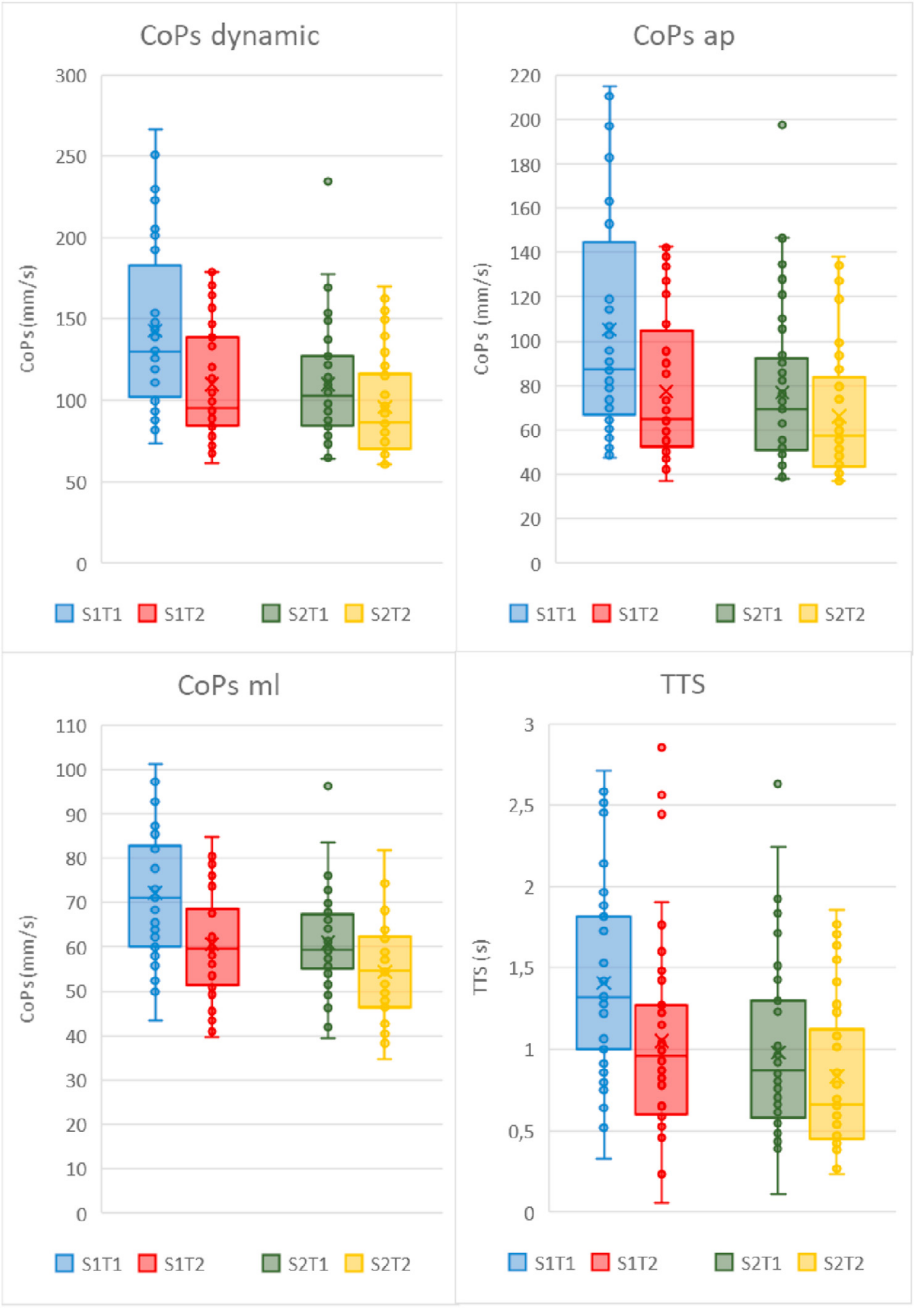
Box plots of the CoPs, CoPs ml, CoPs ap and TTS after platform perturbations for the dynamic tests. Quartiles, medians and individual data points (including outliers) are shown for trials 1 and 2 of session 1 and 2. The x represents the mean in a single session. CoPs = Center of Pressure speed, ml = mediolateral, ap = anteroposterior, TTS = Time To Stabilization, mm = millimeter, s = seconds. S = Session and T = Trial, with for example S1T1 = Session 1 and Trial 1.

The dynamic tests showed higher mean values (i.e., worse performance) for trial 1 compared to trial 2 in both sessions (all p < 0.01; Table 3 and Figure 4). Additionally, an interaction (range p < 0.01 to p = 0.03) was found between session and trial for all the dynamic outcome measures, indicating a larger improvement from trial 1 to 2 in session 1 than in session 2. Furthermore, for each of these variables a higher value was found for session 1 compared to session 2 in the CoPs, CoPs ml, CoPs ap, TTS (range difference 13.2%–26.2%, all p < 0.01) and imbalance (difference 63.2%, p < 0.01).

**Table 3:** Repeated measures ANOVA overall effects and interaction effects (p < 0.05 are indicated with an asterisk).

Outcome measures	Session	Trial	Sex	S x T	S x Sex	T x Sex	S x T x Sex
CoPs static	0.09	0.84	0.02∗	0.15	0.15	0.74	0.95
CoPs dynamic	<0.01∗	<0.01∗	0.57	<0.01∗	0.08	0.45	0.99
CoPs ml	<0.01∗	<0.01∗	0.66	0.03∗	0.13	0.87	0.49
CoPs ap	<0.01∗	<0.01∗	0.61	<0.01∗	0.09	0.43	0.79
TTS_1s	<0.01∗	<0.01∗	0.09	<0.01∗	0.37	0.64	0.97
Imbalance	<0.01∗	<0.01∗	0.83	<0.01∗	0.71	0.21	0.96

S = session, T = trial, CoPs = Center of Pressure speed, ap = anteroposterior, ml = mediolateral, TTS = Time To Stabilization.

**Figure 5:**
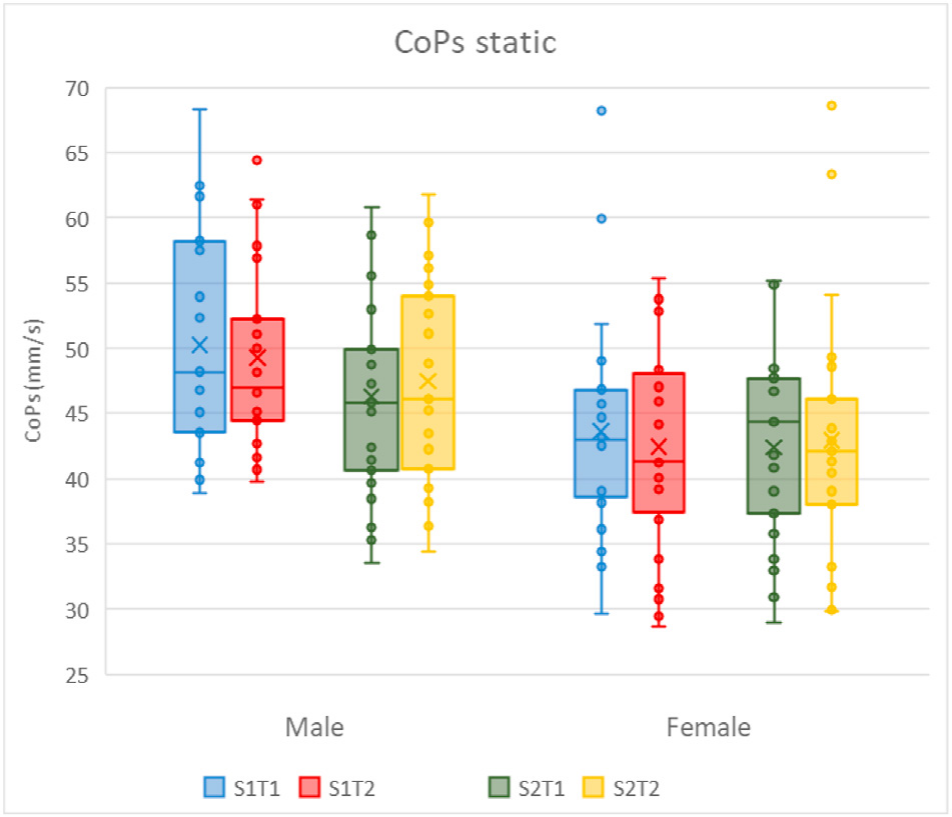
Box plots of the CoPs for the static test for males and females. Quartiles, medians and individual data points (including outliers) are shown for trials 1 and 2 of session 1 and 2. The x represents the mean in a single session. CoPs = Center of Pressure speed, mm = millimeter, s = seconds. S = Session and T = Trial, with for example S1T1 = Session 1 and Trial 1.

In the static tests, females had 11.4% lower CoPs values than males (p = 0.02; Figure 5). The other outcome measures were not significantly different between males and females.

## 4 Discussions and implications

The main finding of this study is that all outcome measures of single-leg dynamic postural stability performance using standardized horizontal platform perturbation tests resulted in fair between-session reliability. To achieve this, we imposed a relatively high number of perturbations (i.e., in each session two trials, each comprising 12 perturbations per leg). This reliability is in line with a similar testing procedure using horizontal platform perturbations in the medio-lateral and anterior-posterior directions [38]. CoPs and TTS correlated strongly (r = 0.66), which underlines the internal validity, but shows that these outcomes still contain substantial independent information about dynamic postural stability. Additionally, the CoPs dynamic and TTS correlated moderately and weakly with CoPs static (r = 0.54 and r = 0.39, respectively), which supports the concurrent validity. On the other hand, this underscores that dynamic postural stability is not equivalent to static postural stability, in line with previous results [18, 22].

In the dynamic tests, we found a decrease of postural stability losses and improvements in CoPs and TTS between trails and sessions, indicating a substantial learning effect. Similar learning effects were found in a variety of dynamic balance tests [26], and when using a balance board [39]. Another study using horizontal platform perturbations found a learning effect only in intra-day comparisons and only for anterior-posterior translations, but not for medio-lateral translations and anterior-posterior translations between sessions [38]. The learning effect in our study was most apparent within session 1. No learning effect was observed for the SLS test. The difference in learning between the static and dynamic tests is likely explained by the difficulty and unfamiliarity of the dynamic task. Since the first trial was significantly different from the other 3 trials, for practical purposes, one might opt to use one trial as a learning procedure. A test session would then consist of 3 trials: one practice trial and 2 trials to assess postural stability. This would also reduce the number of discarded perturbations due to postural stability loss, which will bias the average CoPs value in the direction of better performance. Note that we reported ICC consistency, which is not sensitive to the systematic changes due to learning.

The reliability of CoPs within sessions (ICC = 0.81–0.92) was higher than previously reported for dynamic postural stability outcomes using drop landings [23, 24, 40] and comparable to SLS outcomes [18, 33, 41]. Note that the ICC single between sessions was lower than within sessions, which is expected due to the additional variance in status of the participants between sessions. Compared to the present results, TTS assessed in single leg drop jumps during field testing of youth soccer players was substantially less reliable with ICC values reaching a poor 0.6 [24]. The higher reliability in the present study could be caused by the larger number of repetitions, along with the invariant, externally imposed perturbations. Other studies using body weight to calculate the threshold of the TTS, showed ICC values ranging from 0.64 [42] and 0.69 [37] in two studies using 3 single leg jump repetitions to 0.83 in a study using a single drop step with 10 repetitions [43]. A recent study described a new computational method with a timescale (frequency) approach using wavelet transformation to obtain the TTS [44]. The authors used a large number of repetitions (30), comparable to the present study. Using this new method, ICC values were shown to be higher compared to previously reported methods [24, 44]. However, ICC values were probably inflated relative to our study as these were based on odd versus even landings within a session rather than between session comparisons.

The limited correlation between TTS and CoPs suggests that these outcome measures might reflect different aspects of dynamic stability. A possible explanation for this finding might be that the TTS, which depends on the fluctuation of the vertical forces, is determined by the movement of larger body parts, which would occur when using a hip strategy or arm movements for control of postural stability [45, 46]. The CoPs could be more reflective of the ankle strategy in which small movements in the ankle joint control the center of mass. To validate this explanation, further research is necessary.

For the CoPs analysis of the dynamic postural stability tests we used a window of 2 s after the horizontal platform perturbations. These 2 s were an arbitrary choice, based on figures like Figure 2 which showed a substantial decrease of CoPs within this window. To check the sensitivity of our findings to this choice, we additionally calculated the ICC value of the CoPs for six different time windows (1, 2, 2.5, 3, 4, and 4.8 s) post-perturbation. This resulted in fairly small changes of the ICC values of about 0.01.

The outcome measure Imbalance showed a high reliability between and within sessions. On the other hand, it was not significantly correlated to the CoPs and TTS outcome measures. A possible explanation for this unexpected outcome is that some of the participants consistently chose to use the other leg to recover their postural stability earlier than others.

CoPs and TTS were sufficiently reliable in assessing postural stability after platform perturbations in soccer players. In the clinical setting, the test procedure could be used to evaluate the progress of postural stability during rehabilitation after an injury. This study shows that it is feasible to obtain a high number of repetitions of standardized perturbations within a short measurement time. As a consequence, better reliability can be obtained as compared to drop landing protocols [24]. Compared to recent work on drop landing [44], we not only assessed within session, but also between session reliability. For application in clinical or training assessment, between session reliability is most relevant.

A limitation of the DynSTABLE is that horizontal forces are not measured. This may result in small errors in CoP values. More importantly, outcome measures based on these forces, cannot be calculated. Parameters calculated from horizontal forces, such as TTS and absolute average forces, could provide relevant information on postural stability [47, 48]. Another limitation of this study was the limited sample size (n = 42), although this was larger than in a recent study assessing reliability of drop landing [44]. Nevertheless, a larger sample would provide more precise estimates of the reliability of the platform perturbations. The analysis of the static stability a trial consisted of only 30 s for each leg compared to approximately 5 min of testing for the dynamic stability. Expanding static measurements to a similar amount of time would probably increase reliability.

In this study, we did not match competition level between male and female soccer players. The reason was that levels of competition may be hard to compare between sexes. Furthermore, the initial testing leg was always the left leg, therefore we could not compare between legs. Randomization of the initial testing leg could have provided more information on the dynamic postural control, in that dominant and non-dominant legs could have been compared. However, as our goal was to assess the reliability of postural responses to platform perturbations, and compare this to static single leg stance, we averaged over legs and non-randomizing of the starting leg is unlikely to have had substantial impact on our findings. In addition, we provided real-time feedback based on the CoPs during testing in, to increase awareness of the goal of the test. We do not know to what extent this affected participants’ motor behavior.

## 5 Conclusion

In conclusion, CoPs and TTS outcome measures after standardized horizontal platform perturbations were sufficiently reliable in a sample of soccer players to distinguish a deviant level of postural stability. TTS and CoPs outcome measures during the dynamic tests showed only moderate to strong correlations, suggesting these measures might contain distinct information. In addition, outcomes of the dynamic test were weakly correlated to outcomes of static tests, indicating added value of dynamic tests. Further (clinical) testing of the utility of these outcomes in assessing dynamic balance and their potential relation with underlying postural stability impairments and strategies is indicated.

## Declarations

### Author contribution statement

Paul E. Beelen amd Arnold Huurnink: Analyzed and interpreted the data; Contributed reagents, materials, analysis tools or data; Wrote the paper.

Ricardo Okhuijsen: Conceived and designed the experiments; Performed the experiments; Analyzed and interpreted the data.

Maarten R. Prins, Jaap H. van Dieën and Idsart Kingma: Conceived and designed the experiments; Analyzed and interpreted the data; Contributed reagents, materials, analysis tools or data; Wrote the paper.

Tim Hordijk and Edwin A. Goedhart: Conceived and designed the experiments; Performed the experiments.

Christiaan Kruiswijk and Peter van der Wurff: Conceived and designed the experiments; Analyzed and interpreted the data.

Peter A. Nolte: Analyzed and interpreted the data.

### Funding statement

This research did not receive any specific grant from funding agencies in the public, commercial, or not-for-profit sectors.

### Data availability statement

Data will be made available on request.

### Declaration of interests statement

The authors declare no conflict of interest.

### Additional information

No additional information is available for this paper.
